# Supplementary material for: Salinity Stress-Mediated Suppression of Expression of Salt Overly Sensitive Signaling Pathway Genes Suggests Negative Regulation by AtbZIP62 Transcription Factor in Arabidopsis thaliana
Source: Int J Mol Sci. 2020 Mar 3;21(5):1726. doi: 10.3390/ijms21051726 (PMC7084470; doi:10.3390/ijms21051726)
Supplement: Supplementary file 1 [file ijms-21-01726-s001.pdf]

Salinity Stress-Mediated Suppression of Expression of Salt Overly Sensitive Signaling Pathway Genes Suggests Negative Regulation by *AtbZIP62* Transcription Factor in *Arabidopsis thaliana*

Nkulu Kabange Rolly [1,2], Qari Muhammad Imran [1], In-Jung Lee [3] and Byung-Wook Yun [1,*]

[1] Laboratory of Plant Functional Genomics, School of Applied Biosciences, Kyungpook National University, 702-701 Daegu, Republic of Korea; rolly.kabange@gmail.com (N.K.R.); mimranbot@gmail.com (Q.M.I.); bwyun@knu.ac.kr (B.W.Y.)

[2] National Laboratory of Seed Testing, National Seed Service, SENASEM, Ministry of Agriculture, Kinshasa, 904KIN1, DR Congo

[3] Laboratory of Crop Physiology, School of Applied Biosciences, Kyungpook National University, Daegu, 41566, Republic of Korea; ijlee@knu.ac.kr

* Correspondence: bwyun@knu.ac.kr; Tel.: +82539505712

Received: 07 February 2020; Accepted: 01 March 2020; Published: date

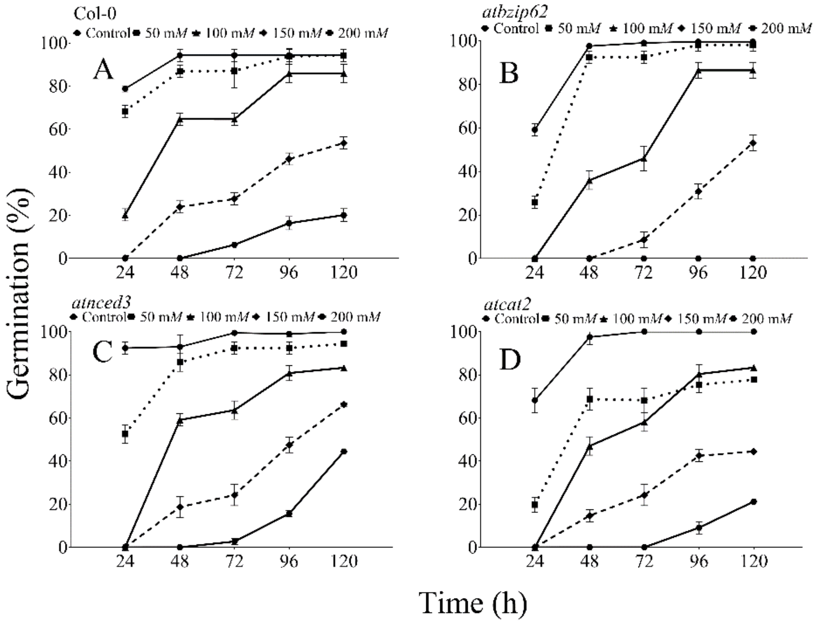

Figure S1 Arabidopsis genotypes evaluated for their germination ability on NaCl modified media
A Germination of Col-0 wild type on different salt concentration overtime. **B** *atbzip62*, **C** *atnced3*, and **D** *atcat2* under the same conditions. Data are mean values of triplicate recorded on daily basis from 24h to 120 h after sowing.

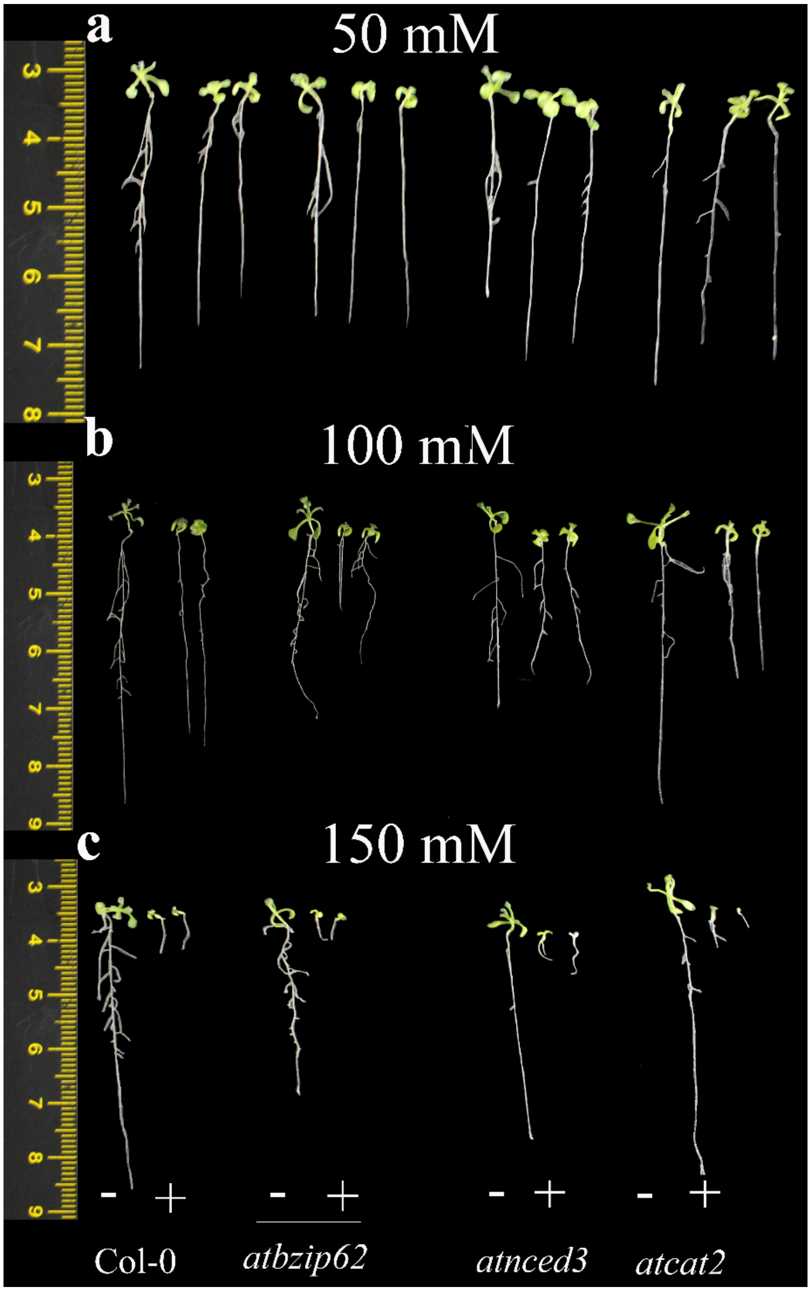

Figure S2 Phenotype of Arabidopsis Col-0 and knockout lines on gradient NaCl concentrations on MS media

The above Figure S2 shows the phenotype of Col-0 wild type, *atbzip62*, *atnced3*, and *atcat2* loss of function mutants in response to salt stress induced by gradient concentrations. **a** 50 m*M*, **b** 100 m*M*, and **c** 150 m*M*. Pictures were taken 10 days after sowing on MS media. Minus (-) sign represents control, and positive (+) sign indicates salt treatment.

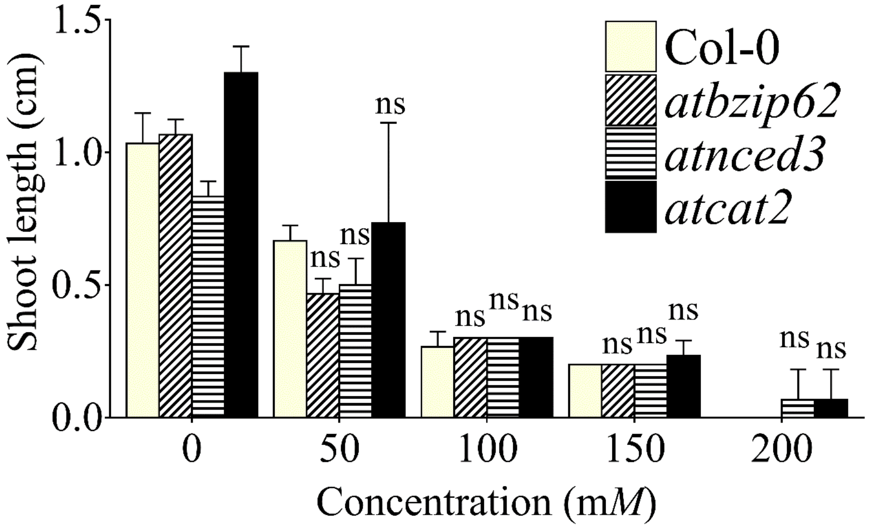

Figure S3 Salt concentration-dependent inhibition of Arabidopsis shoot growth

Figure S3 shows the assessment shoot growth of *Arabidopsis* genotypes (Col-0: wild type, *atbzip62*, *atnced3* and *atcat2*) toward salinity stress on half-strength MS medium gradients concentrations (50, 100, 150 and 200 m*M* NaCl). Data points are the mean of triplicate while error bars indicate ±SD recorded two weeks after sowing. Data are compared with Col-0 wild type. Asterisks on top of bars are statistical significance level with 95% confidence level. *$P<0.05$, **$P<0.01$, ***$P<0.001$, and *ns* non-significant. Error bars are means ± SD.

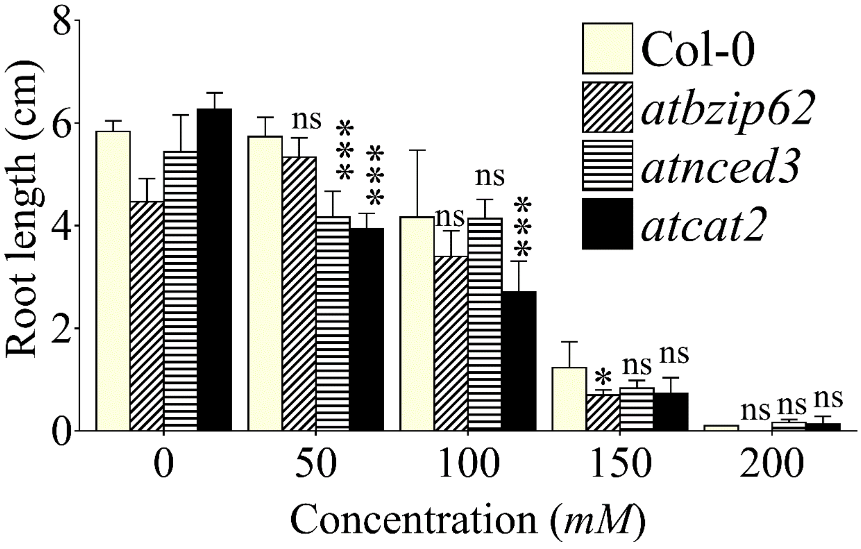

Figure S4 Inhibition of root growth by increasing NaCl-induced salt stress

Figure S4 shows the assessment root growth of *Arabidopsis* genotypes (Col-0: wild type, *atbzip62*, *atnced3* and *atcat2*) toward salinity stress on MS medium gradients concentrations (50, 100, 150 and 200 m*M* NaCl). The data points are the mean values of triplicate while error bars indicate ±SD recorded two weeks after sowing. Data are compared with Col-0 wild type. Asterisks on top of bars are statistical significance level with 95% confidence level. **P<0.05*, ***P<0.01*, ****P<0.001*, and *ns* non-significant. Error bars are means ± SD.

Table S1 Genes for qPCR used in the study

**Gene Name**	**Locus**	**5'-Forwad primer -3'**	**5'-Reverse primer -3'**	**Gene Name**
Drought responsive genes in Arabidopsis				
AtbZIP62	AT1G19490	CATCGAGTTGTTGCTCGTCG	AAATCCGCCAATGCTTCTGC	Basic-leucine zipper transcription factor 62
AtbZIP18	AT2G40620	GACCTCGACACAGGCATAGC	AGCTCAGCGAGCTTATCAGG	Basic-leucine zipper transcription factor 18
AtbZIP69	AT1G06070	GCCATCAGAACCGACTTGGA	CGATTGGCTGTGTTGGTGTC	Basic-leucine zipper transcription factor 69
AtPYD1	AT3G17810	AGTGAGGATCGCTCGCTTTC	TCATCACACCGGTGCATACC	*PYRIMIDINE 1*
Salt Overly Sensitive pathway genes				
AtSOS1	AT2G01980	TTACCAGCCCCCAAGAAACG	TCAACTGTAGGCCAGTCAGC	Arabidopsis Salt Overly Sensitive pathway gene 1
AtSOS2	AT5G35410	TTCAAGACAAGGCTCGAGGG	CCATGAAAAGCGATGGTGCC	Arabidopsis Salt Overly Sensitive pathway gene 2
AtSOS3	AT1G19490	GCAATGGTGTTCCTTTCGGG	GGCGGCATCAGTGTAACTCC	Arabidopsis Salt Overly Sensitive pathway gene 3
AtGLS1	AT5G04140	CTTCTGCATGGGCGACGATA	CCTAAGGGGGTCAATGGCAG	Arabidopsis Glutamate Synthase 1
AtGLS2	AT2G41220	GCAGCATTTAGCCAACCGTC	AGGCTCAACCTTCCCAACAG	Arabidopsis Glutamate Synthase 2
OsACT2	AT3G18780	AGGTTCTGTTCCAGCCATC	TTAGAAGCATTTCCTGTGAAC	Arabidopsis Actin coding gene 2
